# Daily Fermented Whey Consumption Alters the Fecal Short-Chain Fatty Acid Profile in Healthy Adults

**DOI:** 10.3389/fnut.2020.00165

**Published:** 2020-09-30

**Authors:** Nicola M. Smith, Niamh G. Maloney, Sophie Shaw, Graham W. Horgan, Claire Fyfe, Jennifer C. Martin, Andy Suter, Karen P. Scott, Alexandra M. Johnstone

**Affiliations:** ^1^School of Medicine, Medical Sciences and Nutrition, The Rowett Institute, University of Aberdeen, Aberdeen, United Kingdom; ^2^Centre for Genome Enabled Biology and Medicine, University of Aberdeen, Aberdeen, United Kingdom; ^3^Biomathematics & Statistics Scotland, University of Aberdeen, Aberdeen, United Kingdom; ^4^Bioforce AG, Roggwil, Switzerland

**Keywords:** fermented whey concentrate, microbiota, short chain fatty acids, dietary supplementation, postbiotic

## Abstract

Gut microbiota influences many aspects of host health including immune, metabolic, and gut health. We examined the effect of a fermented whey concentrate (FWC) drink rich in L-(+)-Lactic acid, consumed daily, in 18 healthy men (*n* = 5) and women (*n* = 13) in free-living conditions.

**Objective:** The aims of this 6-weeks pilot trial were to (i) identify changes in the gut microbiota composition and fecal short chain fatty acid (SCFA) profile, and (ii) to monitor changes in glucose homeostasis.

**Results:** Total fecal SCFA (mM) concentration remained constant throughout the intervention. Proportionally, there was a significant change in the composition of different SCFAs compared to baseline. Acetate levels were significantly reduced (−6.5%; *p* < 0.01), coupled to a significant increase in the relative amounts of propionate (+2.2%; *p* < 0.01) and butyrate (+4.2%; *p* < 0.01), respectively. No changes in the relative abundance of any specific bacteria were detected. No significant changes were observed in glucose homeostasis in response to an oral glucose tolerance test.

**Conclusion:** Daily consumption of a fermented whey product led to significant changes in fecal SCFA metabolite profile, indicating some potential prebiotic activity. These changes did not result in any detectable differences in microbiota composition. *Post-hoc* analysis indicated that baseline microbiota composition might be indicative of participants likely to see changes in SCFA levels. However, due to the lack of a control group these findings would need to be verified in a rigorously controlled trial. Future work is also required to identify the biological mechanisms underlying the observed changes in microbiota activity and to explore if these processes can be harnessed to favorably impact host health.

**Clinical Trial Registration**: www.clinicaltrials.gov, identifier NCT03615339; retrospectively registered on 03/08/2018.

## Introduction

Over the past decades, an increasing amount of evidence has been emerging on the importance of the gut microbiota driving many biological functions that are directly associated with host health (1). This has subsequently led to an interest in understanding mechanisms and to potentially modulating the microbiota for health benefits.

Within a healthy individual, the intestinal microbiota is reasonably stable in composition, but there is potential for targeted modification toward a more favorable community composition to optimize health benefits. Human intervention studies have shown a clear link between dietary intake and the gut microbiota, where the species composition will adapt in response to dietary change, determined by competition for substrates and gut conditions (2, 3). Therefore, dietary intake is considered the primary modulator of long-term microbiota composition. This association has been observed in long-term prospective studies. These longitudinal data contribute to the increasing causal evidence for the role of the gut microbiota and its metabolites (including short-chain fatty acids, SCFA) on various clinically important indicators of health, such as immune cell function and gut barrier integrity (4–7). In addition, evidence from human intervention studies targeting modification of the microbiota has shown encouraging effects on systemic inflammation, metabolic health, allergies, and a variety of gastrointestinal diseases (8–15). The most salient evidence for the direct link between the gut microbiota and host health comes from the overwhelming success of invasive fecal microbiota transplants (FMT) in the treatment of recurrent *Clostridium difficile* infection (16). This approach focuses on correcting the underlying gut microbiota dysbiosis via drastic changes in the microbiota by replacing host microbiota with those of a healthy donor.

For less invasive approaches, specific foods and dietary patterns can also favorably influence the gut microbiota and metabolite production. In specific instances these health effects have been attributed to prebiotics. A prebiotic is defined as a “selectively fermented ingredient that results in specific changes in the composition and/or activity of the gastrointestinal microbiota, thus conferring benefit(s) upon host health” (17, 18). Prebiotic supplementation can affect the composition and the activity of the gut microbiota (19, 20). Much of the research on these compounds has focused on dietary fibers (particularly oligosaccharides) but other non-carbohydrate compounds, mainly derived from plant materials as well as polyphenols, have been investigated as naturally-occurring prebiotics (21, 22). An example are cocoa-derived flavanols since they are reported to stimulate lactic acid bacteria *in vivo* and *in vitro* in healthy volunteers (*n* = 22) consuming them daily for 4 weeks (23). Further research has shown that lactic acid bacteria in the human gut can convert phenolic compounds, such as flavonoids to biologically active metabolites, and it is believed that these metabolites confer the health benefits to host health. Consumption of a fermented milk product over a 4-weeks period, increased the production of bacterial metabolites, such as butyrate and other short chain fatty acids (SCFAs) in participants with irritable bowel syndrome (IBS-Constipation type Rome III criteria). Patients (*n* = 28) also experienced improvements in self-reported symptoms (i.e., abdominal distention, acceleration of colonic transit time), further strengthening the role of this fermented milk in improving gut health (24). The study authors suggest that these findings were compatible with the observation that butyrate (a short chain fatty acid) has previously been shown to also improve intestinal motility and visceral sensitivity in IBS-C (17, 18, 25, 26). Consequently, renewed efforts are investigating fermentation-derived metabolites in foods, assessing their ability to favorably modulate microbiota composition and activity, thereby acting as potential prebiotics.

Fermented foods are produced through controlled microbial growth, and these food and drink products have recently surged in popularity due to their proposed health benefits (27). Yet, there is limited evidence on the real-world effectiveness of most commercially available fermented food and beverages on gastrointestinal health. Several studies have shown that microorganisms from fermented foods can reach the gastrointestinal tract, in the same way as probiotics which are considered “live microorganisms that, when administered in adequate amounts, confer a health benefit on the host” (28). Studies however indicate that the presence of probiotic species of bacteria appears to be transient when consumption is discontinued in animal and human studies (29, 30).

At present, there is insufficient clinical evidence to make evidence-based recommendations for the consumption of fermented foods to improve the gut microbiota profile. A recent critical review by Stiemsma et al. identified 19 human interventions using fermented foods without probiotic bacterial addition post-fermentation. The heterogeneity in the findings, largely attributed to the variety of foods investigated, highlighted the need to study the bioactive compounds and other beneficial by-products of fermentation individually to ascertain their relative role in mediating health effects (31).

In this study we evaluate a deproteinised whey concentrate, high in L-(+)-Lactic acid, fermented by a member of the genus *Lactobacillus*, establishing its potential as a putative prebiotic. The main aim was to investigate the effect of daily consumption of the whey product on fecal short chain fatty acid composition, on gut microbiota metabolism and composition, and determine if these could be linked to improvement in glycemic control. We also explored the potential underlying physiological mechanisms via assessment of self-reported intestinal function (regularity and consistency of bowel movements).

## Materials and Methods

### Study Design

**Figure 1:**
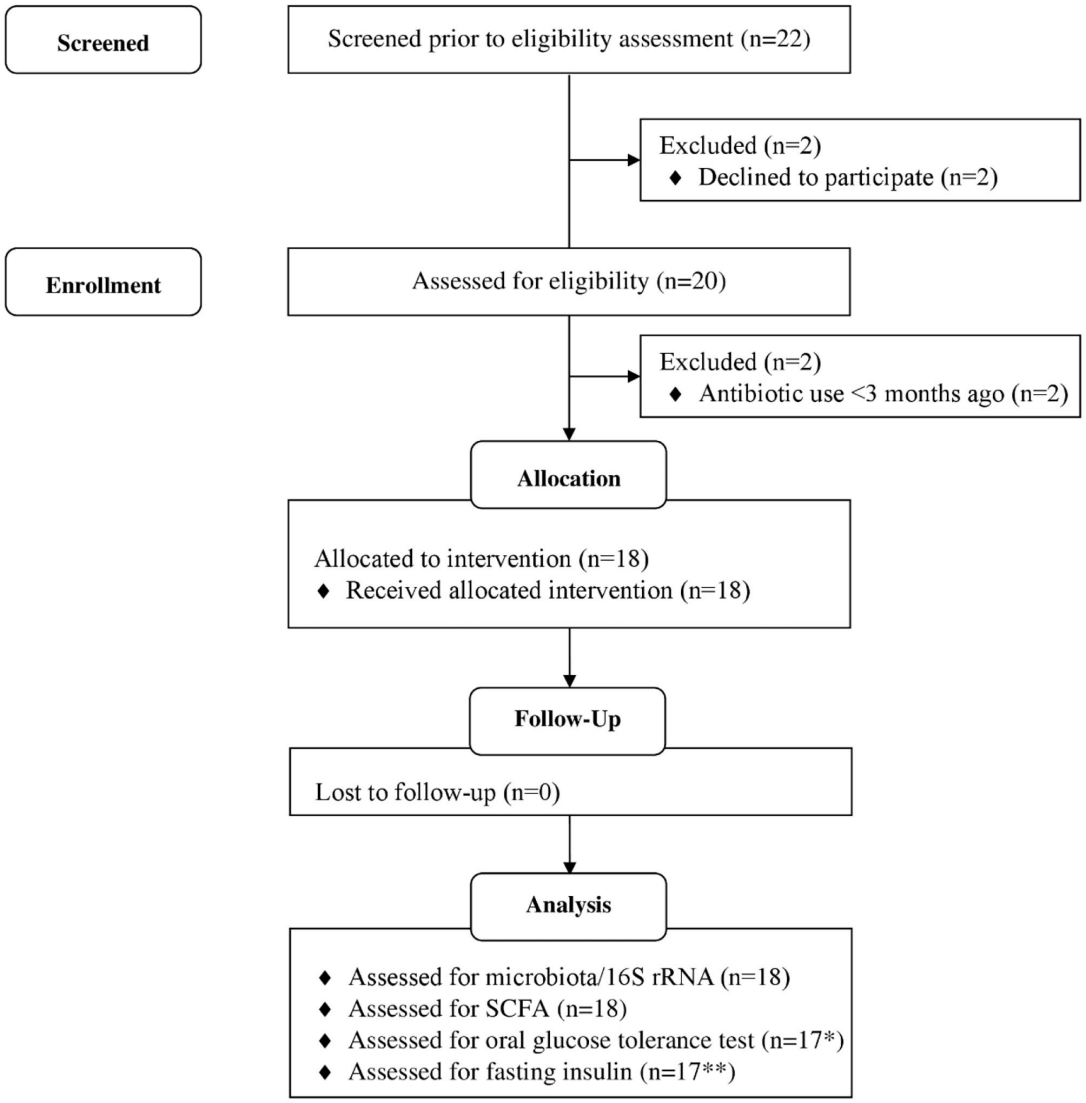
Flow diagram of study participants. *1 participant excluded, as blood samples could not collected due to fainting. **6 participants data excluded as values below threshold of detection of kit. Adapted from (32).

**Figure 2:**
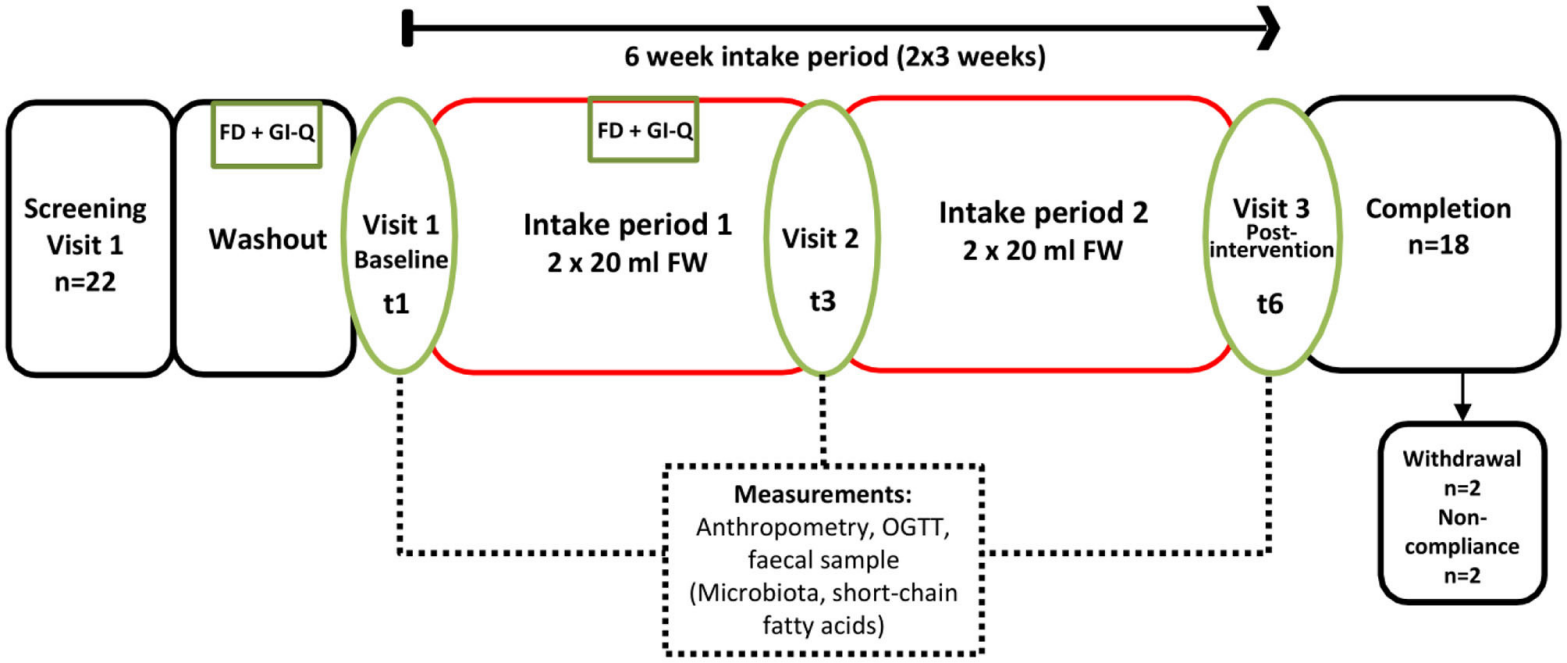
Study Flow (6 weeks continuous intake), single arm intervention, with three separate study visits at the Human Nutrition Unit (referred to as t1, t3, and t6). FD, 3-days food diary; GI-Q, gastrointestinal questionnaire; OGTT, oral glucose tolerance test.

The study was a 6-weeks single-arm intervention study, whereby participants acted as their own control. Participants were 18 healthy adults who attended study visits at the Human Nutrition Unit at the Rowett Institute (University of Aberdeen, UK, Figure 1). Volunteer eligibility was assessed with a health questionnaire during the screening process. The inclusion criteria limited age to 18–65 years and BMI to 18–40 kg/m^2^. Any chronic, uncontrolled disease or use of antibiotics within the last 3 months were deemed exclusion criteria. Enrolled participants were instructed to cease consumption of any probiotics/prebiotics or other food supplements 2-weeks prior to study commencement (washout period) and during the entire intervention. At baseline (t1) and completion (t6), blood pressure, heart rate, and weight measurements were taken (Figure 2).

Blood pressure monitored with the use of an automated system (Omron M5-1; Omron Healthcare Inc., Bannockburn, IL), the average of 3 measures taken was recorded in the supine position.

Height of the participants was measured to the nearest 0.1 cm using a stadiometer (Holtain Ltd, Crymych, Dyfed, Wales). The body weight of the participants was measured wearing a pre-weighed dressing gown, to the nearest 100 g on a digital scale (DIGI DS-410, C.M.S. Weighing Equipment Ltd, London, UK).

### Ethics Statement

The study was conducted according to the guidelines in the Declaration of Helsinki and Good Clinical Practice. The study protocol was approved by the research ethics committee of the Rowett Institute, University of Aberdeen (Reference No. 799). All study participants provided written informed consent. The study was retrospectively registered at ClinicalTrials.gov in August 2018 (NCT03615339).

### Fermented Whey Concentrate Intervention

The investigated product, Molkosan® (A.Vogel, Roggwil, Switzerland) is made of deproteinised fermented organic whey (FWC) concentrate rich in L-(+)-Lactic acid (70 g/L) (33). This filter-sterilized concentrate is naturally lactose-free and fermented by a member of the genus *Lactobacillus*. The concentrate was provided to the participants in 1 L bottles to take home, and they were asked to drink 20 ml of the supplement every morning and evening, diluted in ~200 ml water. Participants were provided with 20 ml measuring cups to allow for accurate measuring of this step. Compliance of daily consumption of the supplement was assessed by the return of empty bottles. The daily supplement intake added 8 kcal in energy intake (~400 kcal for the whole study period, more detailed nutritional information is shown in Table 1). Apart from the high lactic acid content, the SCFA profile of the FWC was quantified to reveal low levels of acetate in addition to the lactic acid (see Supplementary Table 2).

**Table 1:** Nutrition information of the fermented whey concentrate, Molkosan®.

**Nutrition information**	**100 ml Molkosan®**	**Daily amount (40 ml)**
Energy	90 kJ/20 kcal	36 KJ/8 kcal
Protein	0 g	0 g
Carbohydrate of which sugar	<0.5 g <0.5 g	<0.5 g <0.5 g
Fat of which saturated	<0.5 g <0.5 g	<0.5 g <0.5 g
Dietary fiber	0 g	0 g
Sodium	0.1 g	<0.1 g
L-(+)-Lactic acid	7.0 g	2.8 g

### Food Diary and Gastrointestinal Questionnaire

Habitual food intake was recorded using a 4-days weighed food diary prior to study commencement. Measurement of food intake was repeated at week 3 of the intervention, just prior to t3 (Figure 2). Participants recorded details of all foods and drinks consumed or leftover (g) using calibrated kitchen scales (Disc Electronic Kitchen Scale 1036, Salter Housewares, UK), which were provided. Nutritional analyses were conducted using the software NetWISP (version 3.0 for Windows, Tinuviel Software, Anglesey, UK) a computerized version of McCance and Widdowson: The Composition of Foods. Participants also completed a brief gastrointestinal symptom questionnaire (GI-Q) and were asked to record the number of bowel movements over the same 4 days. The questionnaire asked participants to record the severity of nausea, bloating, flatulence, cramps and rumbles on a 3 point hedonic scale, ranging from “slightly more symptoms than usual,” “noticeably more symptoms than usual” to “considerably more symptoms than usual” (16).

### Oral Glucose Tolerance Test (OGTT)

Volunteers were requested to fast from 10 p.m. the previous evening prior to study visits on days 0 (t1), 21(t3), and 42 (t6). After baseline blood samples were taken participants were given 113 ml of Polycal (Nutricia Advanced Medical Nutrition, UK), dissolved in 87 mL of water, corresponding to 75 g of anhydrous glucose to drink. An oral glucose tolerance test (OGTT) was then carried out by collecting 5 ml of venous blood at 0, 15, 30, 45, 60, 90, 120, 150, and 180 min post-glucose intake, via a cannula into lithium heparin tubes (S-Monovette®, Cat #04.1936, Sarstedt, Germany). Plasma was analyzed for glucose and insulin concentration [see section Oral Glucose Tolerance Test (OGTT)]. The total area under the curve (tAUC) was calculated using the trapezoid method for (a) the total duration of the OGTT and (b) each incremental area under the curve (iAUC). For any missing data during OGTT, values were imputed based on the mean of the value recorded on the preceding and subsequent time point. Statistics were performed using log-transformed data for a two-way ANOVA using Genstat 17 Release 17.1 (Lawes Agricultural Trust, VSN international Ltd, Hemel Hempstead, UK).

### Blood Sample Analysis

One participant was unable to provide blood samples, so the data presented is for *n* = 17 (94.4%). Biochemical parameters, including glucose, and the fasting lipid profile (total cholesterol, LDL, HDL, non-esterified fatty acids and triglycerides), were measured using a Konelab30 clinical autoanalyser (Kone Limited, Thermo Fisher Limited, USA). Insulin was quantified, in duplicate, by enzyme-linked immunosorbent assay (Mercodia insulin, Mercodia Inc., Sweden) as per manufacturer's instructions. Complete data were available from 12 individuals for both baseline (t1) and end-of-study (t6) insulin levels above the detection threshold of the assay (>2 mIU/L).

### Fecal Sample Processing and DNA Extraction

Participants self-collected stool samples within 16 h preceding their study visits (Fecontainer, AT Medical BV, The Netherlands) and kept cold at 4°C. Fresh feces (5 g) were diluted 1:2 (w/v) with phosphate-buffered saline (pH 7.4, 30% glycerol) and homogenized by gentleMACS Dissociator (Miltenyi Biotec, Bergisch Gladbach, Germany). Aliquots (450 μl) were used for DNA extraction using FastDNA™ SPIN kit for Soil (MP Biomedicals, Santa Ana, USA) and stored at −70°C.

### Short Chain Fatty Acid Quantification

The SCFA content of the samples was determined by capillary gas chromatography following conversion to t-butyldimethylsilyl derivatives. SCFA were quantified in mM against authentic standards of acetate, propionate, butyrate, valerate, and the branched chain fatty acids iso-butyrate and iso-valerate. Samples were analyzed using a Hewlett Packard gas chromatograph fitted with a fused silica capillary column with helium as the carrier gas. The lower limit for reliable detection of each product was taken as 0.2 mM. The method used has been described previously (34).

### 16S rRNA Gene Sequencing

The V1–V2 region of the 16S rRNA gene was amplified by PCR using primers containing adaptors for downstream Illumina MiSeq sequencing. Primers used were 27F and 338R. The pooled PCR amplicons were precipitated and re-suspended in TE buffer (10 mM Tris-HCl, 0.1 mM EDTA). Quantification was performed using the Qubit dsDNA HS Assay Kit and Qubit fluorometer 2.0 (Invitrogen, USA). Prior to sequencing samples underwent purification using AmPure XP beads (Beckman Coulter, USA) Amplicon samples were provided to the Center for Genome Enabled Biology and Medicine (Aberdeen, UK) and sequenced using the Illumina MiSeq v2 flowcell (Illumina, CA, USA) producing 250 bp paired end reads.

### Bioinformatic Analysis

Quality of the sequences was assessed using FastQC (version 0.11.3) and sequence variant abundances were determined using DADA2 (version 1.3.1) (35, 36). Taxonomy was assigned against the GreenGenes 13.8 database (37). The outcome sequence variant was converted to biom format using biomformat (version 2.1.3) (38). Diversity analysis was performed using the core_diversity_analyses.py script from QIIME (version 1.9.0) (39) with subsampling of 6,730 sequences per sample.

Core diversity analyses calculated five alpha diversity measures, including the Shannon Index, and two beta diversity measures, Bray Curtis and Binary Jaccard (40–43). Figures were generated in R using ggplot2 unless otherwise stated. PCoA plots were visualized using EMPeror (44).

Statistical testing of stratification of samples by meta data category was performed using the adonis statistical test on the Bray Curtis diversity metrics, implemented by the compare_categories script from using QIIME (version 1.9.0) (39, 45).

Differential abundance test of sequence variants between volunteers classified responders (*n* = 5, Volunteers 18, 4, 14, 20, 12) vs. non-responders (*n* = 5, Volunteers 7, 15, 11, 21, 6) based on the largest increase in butyrate concentration and repeated for propionate levels (see Supplementary Tables 1A, B). This comparison was carried out using a beta-binomial regression model for abundance data using the R package corncob (46). Comparisons were made at the taxonomic ranks of phyla, family, genus and species at all three time points throughout the study. Sensitivity analyses were run to test the influence of sequence variants present in <5% of samples.

## Results

### Study Group Characteristics and Participant Anthropometry

Of the 22 participants enrolled, 18 completed the study (two were excluded due to antibiotic intake, two due to time constraints; Figure 1). Group characteristics are summarized in Tables 3, 4. Overall, the study group was largely female (72%; *n* = 13) and had an average age of 39 (*SD* = 2.6) years. On average participants weighed 69.2 (*SD* = 13.4) kg, ranging from 49.6 to 95.1 kg at baseline (t1). Blood pressure and heart rate remained in a healthy range throughout the whole study period. Baseline clinical biochemistry measured from blood samples indicated all volunteers were in good health prior to and during the intervention (see Table 2). Although there was a statistically significant increase in mean LDL cholesterol at t6 up to 2.62 mmol/l (see Table 2), this is attributed to a single volunteer experiencing a sharp increase to 5.22 mmol/l. Excluding this volunteer's measurements indicated average changes were non-significant, t6 = 2.46 mmol/l (*n* = 16, *p* = 0.15).

**Table 2:** Blood profile.

	**Cholesterol (mmol/l)**	**HDL Cholesterol (mmol/l)**	**LDL Cholesterol (mmol/l)**	**Total-HDL Cholesterol Ratio**	**LDL-HDL Cholesterol Ratio**	**NEFA^*a*^ (mmol/l)**	**Triglycerides (mmol/l)**
Baseline (t1)	4.53 ± 0.16 (3.57–5.91)	1.52 ± 0.09 (0.85–2.37)	2.42 ± 0.17 (1.43–3.84)	3.19 ± 0.27 (1.89–6.41)	1.77 ± 0.23 (0.66–4.54)	0.55 ± 0.06 (0.24–1.11)	1.19 ± 0.14 (0.56–2.56)
Fermented whey (t6)	4.66 ± 0.18 (3.81–6.73)	1.55 ± 0.10 (0.95–2.45)	2.62 ± 0.22 (1.45–5.22)	3.28 ± 0.32 (1.81–7.11)	1.92 ± 0.28 (0.59–5.51)	0.52 ± 0.06 (0.17–1.26)	1.04 ± 0.11 (0.54–1.80)
Change	0.14	0.03	0.21	0.09	0.15	−0.03	−0.15
% change	3.03	1.93	8.51	2.74	8.64	−5.51	−12.66
*p*-value	ns	ns	**0.05**	ns	**0.04**	ns	ns
log_v_	ns	ns	**0.04**	ns	ns	ns	ns

*Mean ± SEM (Range) (n = 17)*.

a *Non-esterified fatty acids*.

*The bold values are significant p-values*.

**Table 3:** Baseline (t1) anthropometric characteristics including all participants who completed the study (*n* = 18).

**Age (years)**	**Height (m)**	**Systolic BP (mmHg)**	**Diastolic BP (mmHg)**	**Pulse (beats/min)**
39 ± 2.6 (20–55)	1.69 ± 0.02 (1.56–1.92)	117.7 ± 2.9 (98–148)	69.4 ± 2.2 (56–91)	64.8 ± 2.3 (46–83)

*Data shown as mean ± SEM (Range)*.

**Table 4:** Weight and BMI, as measured at initial enrollment and at the completion of the intervention (*n* = 18).

	**Males (*n* = 5)**		**Females (*n* = 13)**		**All (*n* = 18)**	
	**Weight (kg)**	**BMI (kg/m^2^)**	**Weight (kg)**	**BMI (kg/m^2^)**	**Weight (kg)**	**BMI (kg/m^2^)**
Baseline (t1)	76.6 ± 8.1 (59.2–95.4)	24.1 ± 2.70 (18.75–29.75)	67.7± 3.6 (51–90.7)	24.8 ± 1.14 (18.9–32.7)	70.5 ± 3.3 (51–95.4)	24.6 ± 1.0 (18.7–32.7)
Fermented whey (t6)	74.9 ± 8.8 (59.2–98.7)	23.1 ± 2.6 (18.5–30.7)	69.7 ± 2.9 (54.8–91.1)	25.6 ± 1.0 (19.6–32.4)	71.1 ± 2.9 (54.8–98.7)	24.9 ± 0.9 (18.5–32.4)
Mean change	−1.7	−1.0	2.0	0.8	0.6	0.3

*Data shown as mean ± SEM (Range)*.

#### Energy Balance and Diet

Participant BMI did not change over the 6-weeks intervention period. The 4-days food diaries indicated that dietary energy intake and macronutrient composition, on average, remained stable during the intervention (Table 5).

**Table 5:** Average energy intake and macronutrient composition of study participants diets as reported by 4 days weighed food diaries (*n* = 18).

**Energy intake**	**Baseline**	**Intervention**	***p*-Value**
Energy (kJ)	9,106 ± 565 (6,545–16,886)	9,063 ± 546 (6,402–15,571)	ns
Energy (kcal)	2,179 ± 135 (1,566–4,040)	2,168 ± 131 (1,531–3,725)	ns
**MACRONUTRIENTS**			
Carbohydrate (%)	47.6 ± 1.3 (37.3–56.7)	47.2 ± 1.1 (38.8–53.4)	ns
Protein (%)	17.2 ± 1.0 (10.2–24.3)	16.0 ± 0.9 (11.1–21.1)	ns
Fat (%)	31.5 ± 0.95 (24.2–39.2)	32.7 ± 0.9 (25.2–36.8)	ns
Starch (g)	162.9 ± 10.4 (101–283.5)	157.7 ± 9.8 (90.9–230.3)	ns
Fiber (g)	23.9 ± 1.5 (14.4–37.7)	23.8 ± 1.9 (14.0–43.5)	ns

*ns, not significant (p-value > 0.05)*.

*Data shown as mean ± SEM (Range)*.

### Oral Glucose Tolerance Test

**Figure 3:**
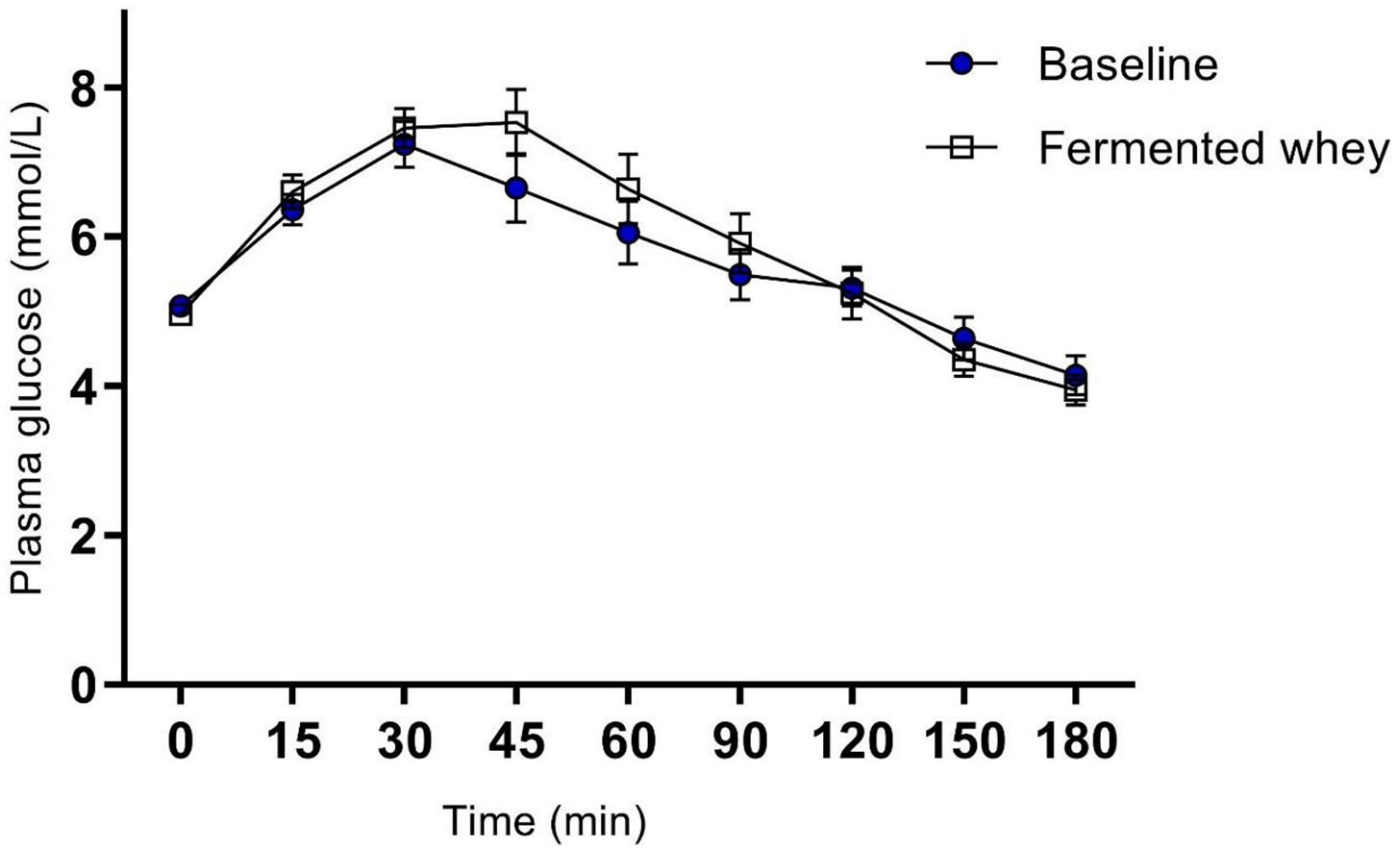
Oral Glucose tolerance test showing group plasma glucose profile in response to a 75 g bolus of glucose, at commencement of study (Baseline) and after 6-weeks of chronic fermented whey consumption (Fermented whey, *n* = 17).

There were no significant changes after the 6-weeks fermented whey intervention in glycemic response in volunteers (*n* = 17) (Figure 3). Fasting blood glucose was comparable before and after FWC consumption (t1 = 5.08 mmol/L, *SD* = 0.38, t6 = 4.96, *SD* = 0.43). In addition, analysis of tAUC (t1 = 1,004, *SD* = 149.5, t6 = 1,032, *SD* = 175.9, *p* = 0.619, *n* = 17) and iAUC (t1 = 148, *SD* = 89.5, t6 = 194, *SD* = 136, *p* = 0.324, *n* = 17) showed these were not significantly influenced by FWC intake.

**Figure 4:**
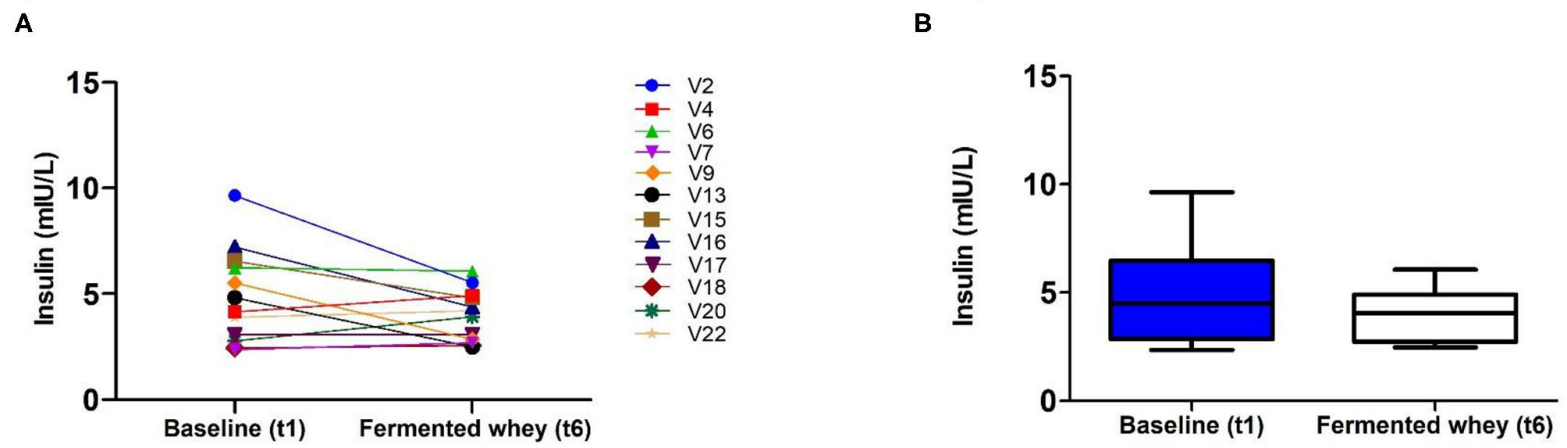
Fasting insulin profiles pre- (t1) and post-intervention, t6 (*n* = 12). **(A)** Insulin concentration shown by individual volunteers, at baseline (t1) and after the intervention (t6). **(B)** Group average fasting insulin levels. There was a reduction of −0.9 mUI/L which failed to reach significance (*p* = 0.089).

Analysis of insulin levels at baseline (t1) and at week 6 (t6), showed strong interindividual variation (Figure 3). Seven of the 12 volunteers had lower levels at t6 and the remaining five showed little change as indicated by the individual insulin responses shown in Figure 4A. On average the participants show no significant change in their fasting insulin levels over the duration of the intervention (t1 = 5.11 mIU/L, SD = 2.08, t6 = 4.075 mIU/L, *SD* = 1.16–0.9 mIU/L; *p* = 0.089, Figure 4B).

### Self-Reported Gastrointestinal Well-being

There was no significant change in the average number of daily bowel movements (1.2–1.3, *n* = 18). Subjective feelings of gut health were assessed from two sets of daily questionnaires completed in parallel with the 4-days food diaries (Table 6). To monitor overall changes, we compared the proportion of responses recorded as normal at baseline with the data collected on day 21 (t3). There was no change from normal in the reported feelings of nausea, flatulence, cramps and rumbles. On the other hand, the proportion of days that participants reported more bloating than usual declined following fermented whey concentrate (FWC) consumption from 22.5% instances to 9.9%.

**Table 6:** Gastrointestinal symptoms were self-reported for 4 days (*n* = 18) just prior to baseline and week 3 of the trial (t3)^a^.

**Scale**	**Baseline symptoms (week-1)**				
	**Nausea**	**Bloating**	**Flatulence**	**Cramps**	**Rumbles**
0—normal	68	55	43	63	49
1—“Slightly more than usual”	1	14	24	7	16
2—“Noticeably more than usual”	2	2	3	1	5
3—“Considerably more than usual”	0	0	1	0	1
% Normal	95.8	77.5	60.6	88.7	69
	**Fermented whey (week 3, t3)**				
0—Normal	67	64	40	64	51
1—“Slightly more than usual”	4	6	28	4	16
2—“Noticeably more than usual”	0	1	3	3	4
3—“Considerably more than usual”	0	0	0	0	0
% Normal	94.4	90.1	56.3	90.1	71.8

*The table summarizes number of responses recorded for each level over 4-days (with 0 indicating “normal” function till 3 considered as “considerably more symptoms than usual”). The percentage normal, refers to the proportion of responses being 0 on the scale*.

a *At both time points t1 and t3, one volunteer only reported 3-days, only reporting 71 observations out of 72*.

### Short-Chain Fatty Acid (SCFA) Analysis

**Figure 5:**
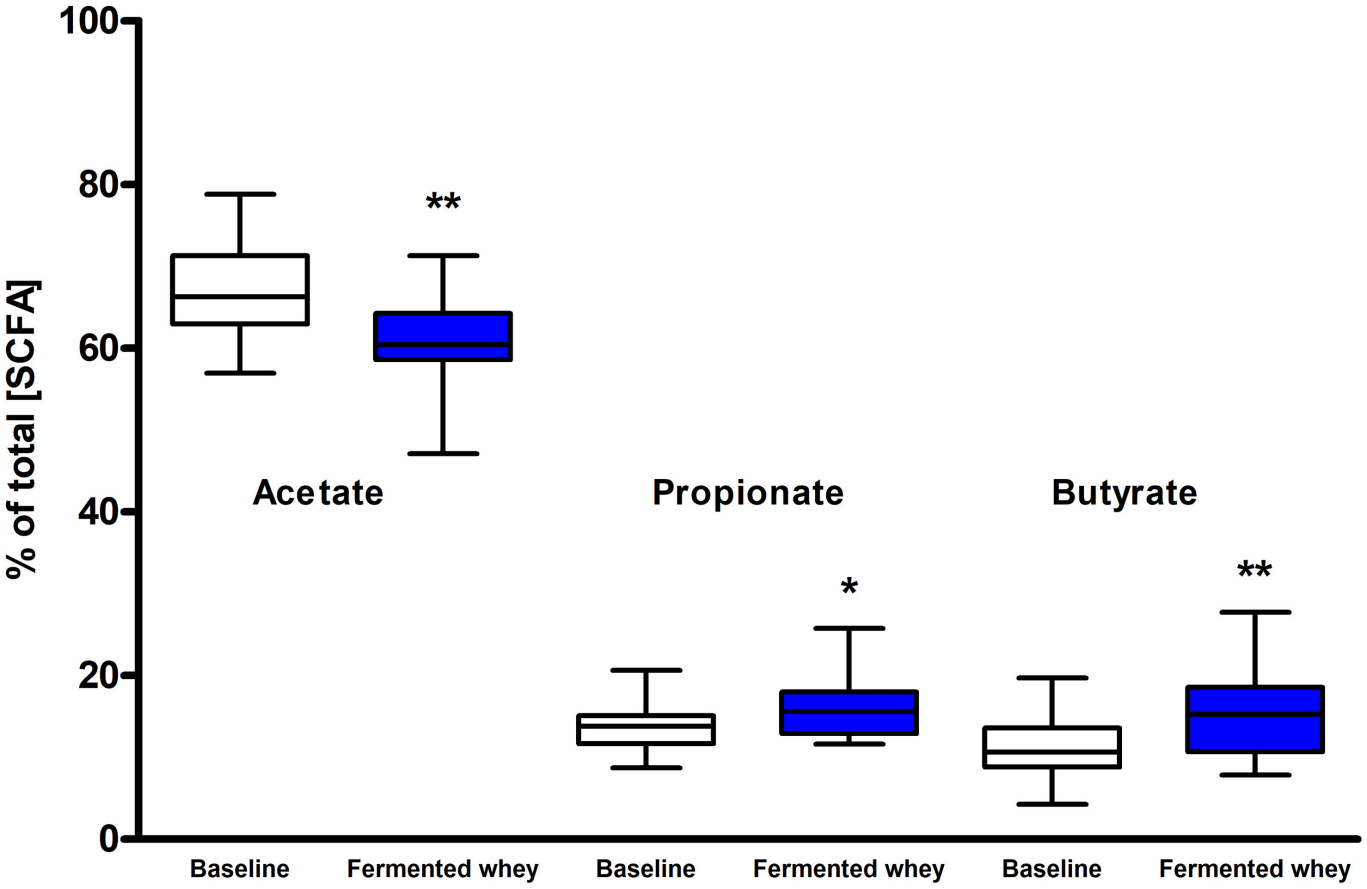
Short chain fatty acid composition of fecal samples (*n* = 18). Values are proportions of total SCFA concentration. Boxplots whiskers represent min/max values. Acetate levels decreased significantly (by 6.48%, *p* < 0.01), while both propionate (*p* < 0.05) and butyrate (*p* < 0.001) levels increased. No lactate or succinate were detected. (* = *P* ≤ 0.05, ** = *P* ≤ 0.01, *** = *P* ≤ 0.001).

SCFA and branched chain fatty acids (BCFA) were quantified to assess the impact FWC (rich in lactate) supplementation on bacterial metabolic activity. The total SCFA + BCFA concentration was relatively constant at each time point (t1 = 88.8 mM, *SD* = 31.8 and t6 = 85 mM, *SD* = 39; *n* = 18). No significant changes were observed in the levels of the BCFA iso-butyrate and iso-valerate, and the SCFAs valerate or the intermediate metabolite succinate (*p* > 0.05, data not shown). Lactate was not detected in any fecal samples (data not shown). There was a significant decrease in the relative proportion of acetate and a concomitant increase in propionate and butyrate observed at week 6 (t6) of the intervention when compared to baseline measurements (Figure 5). Acetate levels were significantly reduced from an average of 66.98%, *SD* = 5.69% at t1 to 60.5%, *SD* = 5.41% (*p* < 0.01) at t6. In parallel a significant increase in the relative amounts of propionate by 2.2% (t1 = 13.63%, *SD* = 2.91; t6 = 15.79%, *SD* = 3.59, *p* < 0.05) and butyrate by 4.2% (t1 = 11.34%, *SD* = 3.94; t6 = 15.55%, *SD* = 5.72, *p* < 0.01), were observed (Figure 5).

### 16S rRNA Gene Sequencing and Microbiota Composition

Raw 16S rRNA gene sequence data generated between 14,299 and 141,814 reads per fecal sample, with an average read count of 62791. Analysis of raw sequencing reads using DADA2 produced a total of 6,377 sequence variants across all samples.

We did not observe any change in overall bacterial α-diversity, measured by Shannon index, over the duration of the study, as illustrated when the samples are grouped together based on time point (Figure 5), suggesting that FWC supplementation does not impact on the number of bacterial taxa within the gut. Looking at relative abundance of taxa, when the samples are grouped by volunteer the underlying inter-individual variation is apparent at both phylum and genus level (**Figure 7**). Clustering of samples based on microbiota composition according to Bray-Curtis dissimilarity indices, showed no separation based on fermented whey consumption (Adonis *p* = 1; **Figure 7**), with any division of samples driven by the individual's microbial composition (Adonis *p* = 0.001; **Figure 8**). In fact, this figure clearly shows that the three samples from each individual cluster strongly together, illustrating that the microbiota within an individual is stable through the duration of the study.

The volunteer group was split into responders (*n* = 5) and non-responders (*n* = 5) according to the highest and smallest change in butyrate levels. There were no significant differences between these two groups in terms of alpha and beta diversity. Since the responder group was based on higher than average detection of butyrate following FWC supplementation, the relative abundance of the following known butyrate-producing species was investigated: *Anaerostipes* species, *Coprococcus eutactus* (Figure 9), *Eubacterium hallii, Eubacterium rectale, Faecalibacterium prausnitzii*, and *Roseburia* species (47), however no differences in abundance of any of these bacterial groups was observed (data not shown). A separate analysis was carried out based on propionate and separately butyrate responders (largest change in proportion of SCFA), compared to non-responders (volunteers with smallest change in levels). Results are summarized in Supplementary Tables 1A,B). For example, some significant differences were observed between other bacterial groups. A family of bacteria classed as *Veillonellaceae*, in the genus *Dialister*, were significantly higher in the butyrate responder volunteer sub-set (FDR = 0.0047; false discovery rate correction for multiple testing) at t3 and t6 (Supplementary Table 1B).

## Discussion

**Figure 6:**
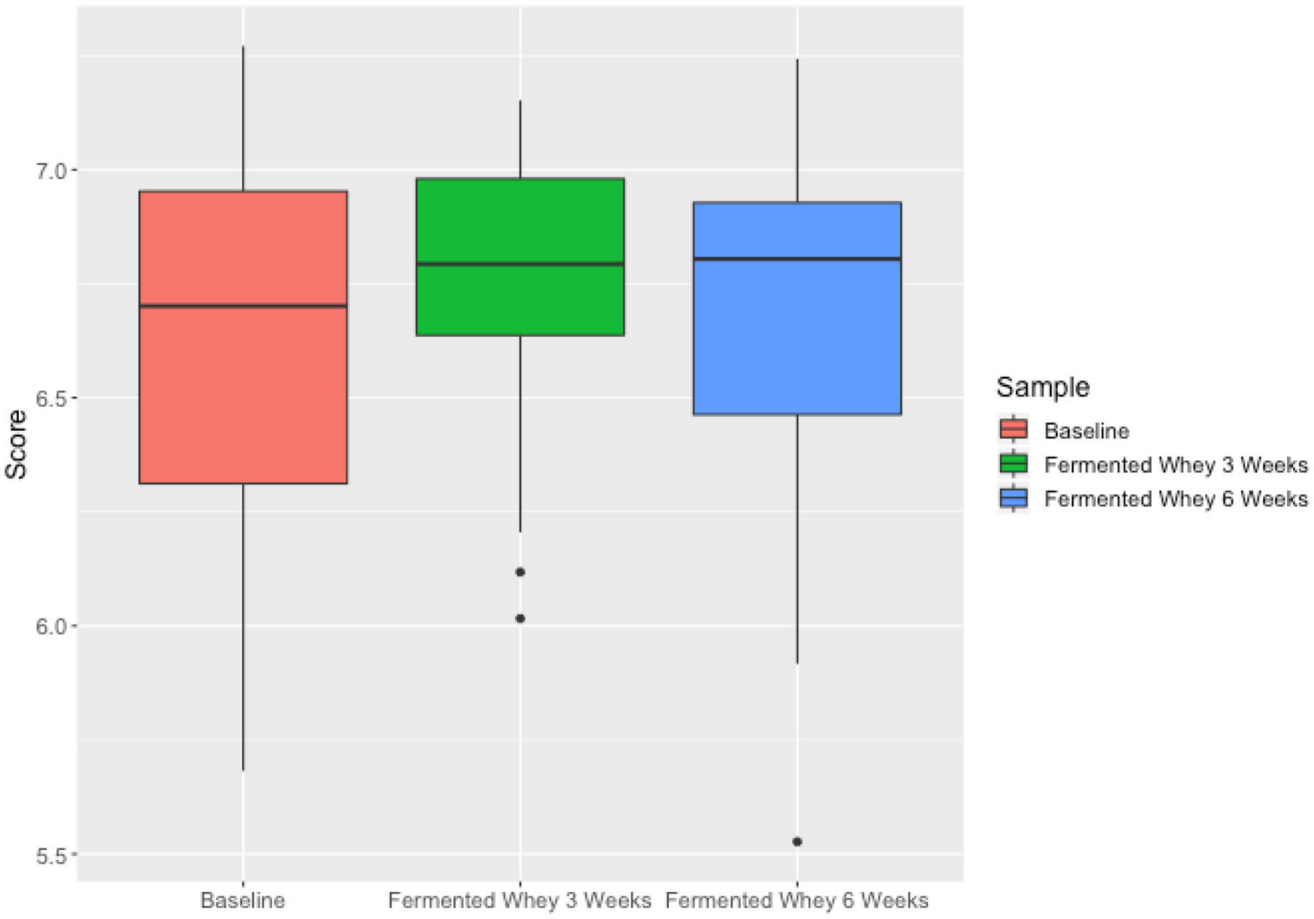
Microbiota alpha diversity metrics, analyzed by calculating the Shannon index comparing samples based on time point (t1—red, t3—green, t6—blue). Differences over time were not significant.

**Figure 7:**
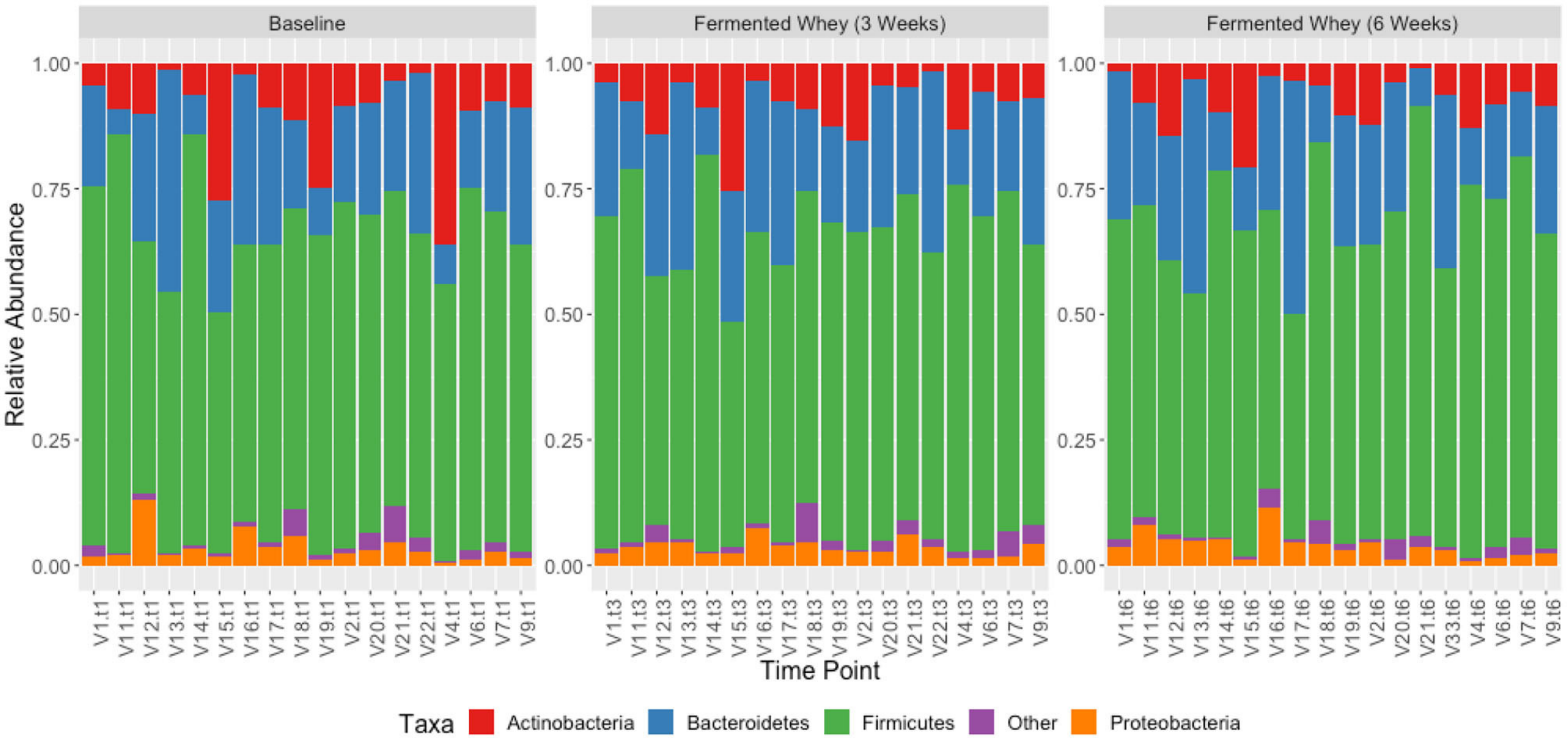
Taxonomic profile of individual volunteers at phylum level, grouped by time-point, t1, t3, and t6.

**Figure 8:**
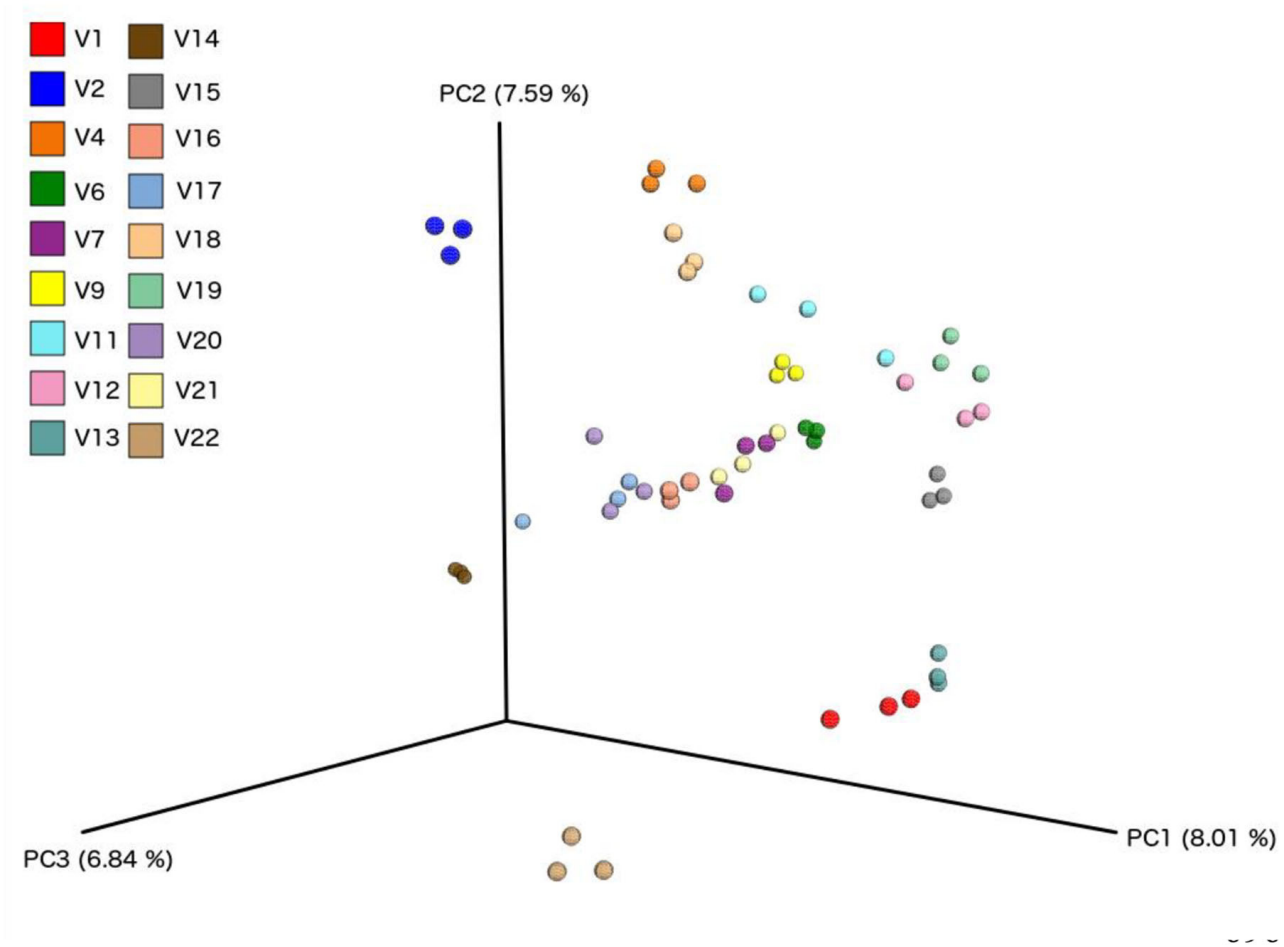
Principle component analysis plots based on Bray-Curtis Dissimilarity Index, comparing each sample from individual volunteers (*n* = 18).

We present the results of a diet study investigating the effects of dietary supplementation with a fermented whey product containing bacterially derived L-(+)-Lactic acid on various health parameters and microbial activity. Following daily FWC supplementation in healthy adults, a higher proportion of butyrate and propionate were detected in fecal samples, although total SCFA levels remained unchanged (Figure 5). This may be attributable to the high L-(+)-Lactic acid content (70 g/L) of the FWC influencing the activity of lactate utilizers within the host microbiota. However, we were unable to detect a significant change in the composition of the microbiota following the addition of FWC to the diet. No significant changes were detected in alpha diversity (Figure 6, Shannon-diversity index), bacterial abundance at the phylum level (Figure 7), or in beta diversity (Figure 8, Bray-Curtis dissimilarity) over the course of the supplementation. This may in part be due to the large inter-individual variability in bacterial species composition which our study was not sufficiently powered to overcome. Further investigations would be needed to determine whether oral ingestion of lactate might stimulate cross-feeding of lactate utilizing species in the colon resulting in production of butyrate or propionate in healthy volunteers (48, 49).

**Figure 9:**
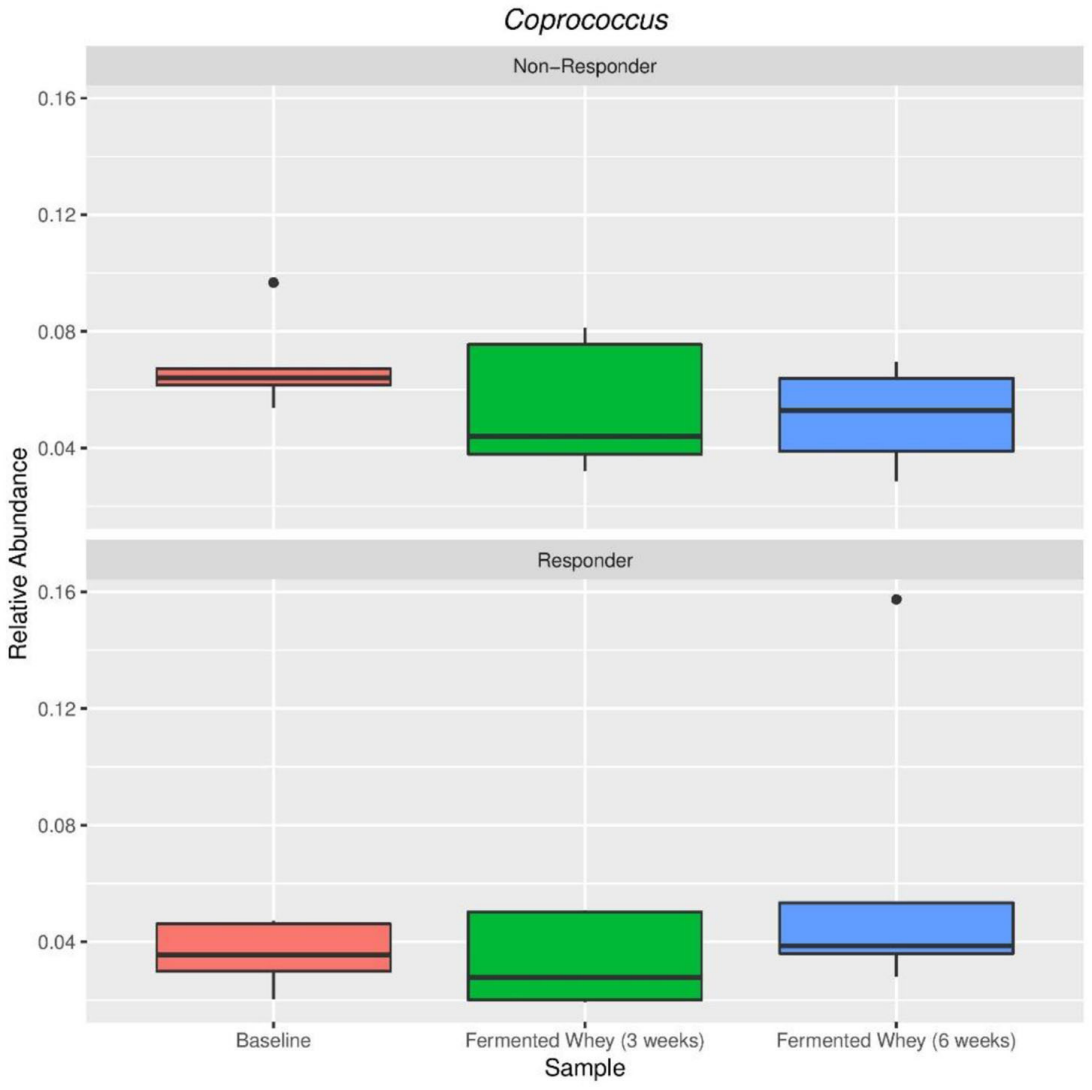
Relative abundance of *Coprococcus eutactus* in volunteers with the highest (Responder, *n* = 5) and lowest (Non-responders, *n* = 5) changes in fecal butyrate concentration. Kruskal-Wallis test show non-significant changes in abundance across sample types in both responders (*p* = 0.38) and non-responders (*p* = 0.38).

Lactate is rarely present in healthy adult feces due to extensive cross-feeding between different bacterial species whereby any lactate produced provides a substrate for lactate-utilizers in the microbiota (1, 43). In this study lactate was not detected in feces, even after supplementation with FWC rich in L-(+)-Lactic acid. This could indicate that the L-(+)-lactic acid was either not able to reach the colon and was absorbed in the upper GI tract, or was completely metabolized by microorganisms (50). There is considerable uncertainty on the biodistribution of orally (exogenous) ingested L-(+)-lactic acid in humans due to the continual flux of naturally occurring lactate from metabolic processes, even at rest (51). Compared to endogenously produced lactic acid, the lactic acid consumed from the FWC will have a substantially different biodistribution, as it is subjected to the multitude of digestive processes all along the human gastrointestinal tract. In particular, the initial stages of exogenous lactate digestion (absorption and distribution) will differ to endogenous lactate, which is directly released from the human cell into circulation (52, 53). In healthy adults, plasma lactate is primarily cleared by the liver and mitochondria-rich tissue, such as skeletal muscle (54, 55).

Exploring the fate of exogenous lactate, produced by bacterial fermentation, within the gastrointestinal tract would improve our understanding of dietary lactic acid intake and the downstream effects on host-microbiota composition, and eventually bioactivity. Commonly consumed fermented foods are naturally rich in L-(+)-Lactic, yogurt can reach up to 2%. This warrants further investigation into the effect of regular ingestion of these levels and whether these contribute to the beneficial health effect associated with regular yogurt consumption (56). The FWC used in our study contains 7% L-(+)-lactic acid (or 70 g/L), meaning that the daily intake of the volunteers corresponded to 2.8 g of L-(+)-lactic acid, comparable with concentrations in a pot of yogurt. It is important to note that this lactic acid was administered independent of any live bacteria or food matrix (in contrast to fermented foods), which may have affected its absorption. It would be interesting to investigate whether regular ingestion of these levels of lactate contribute to the beneficial health effect associated with regular yogurt consumption (56).

A further aspect to consider is that systemic lactate can cross the gut epithelial barrier and thus it is possible that not all FWC lactate directly reached the colon (56). Even if dietary lactate is absorbed into circulation in the upper GIT, at least some of it may reach the colon via indirect mechanisms (systemic circulation and transport back into the colon). A previous study by Scheiman et al., linked the presence of *Veillonella* bacteria, conversion of circulating lactate to propionate, and enhanced athletic performance (57). The results from our study were in line with these previous findings yet it remains unknown if this mechanism of peripheral lactate transport into the GIT can be observed independent of exercise. Our data shows that consumption of FWC [containing L-(+)-lactic acid] increases relative amounts of butyrate produced in feces, but only in participants who had higher levels of *Veillonellaceae* (Dialister) species (see Supplementary Table 1A).

Overall, there were no changes in bacterial diversity as a result of FWC supplementation. Both the alpha diversity (the number of different taxa) and the Shannon index (sample richness) indicated similar bacterial abundance levels across the study period. However, when the data were stratified into *responders* and *non-responders* based on increased fecal butyrate detection, the *Veillonellaceae* family were significantly higher in responders (FDR = 0.0047) at both sampling timepoints. This may indicate that those individuals with a higher resident population of bacteria capable of converting lactate to butyrate/propionate, have higher concentrations of these SCFA in feces following lactate consumption. Consequently, the modified SCFA profile indicates that the lactic acid might stimulate microbial metabolic activity without detectable changes in microbial composition. Metatranscriptomic analysis of the microbiota would show which genes were differentially expressed by bacterial species following FWC supplementation and provide detailed insights into changes in metabolic activity. Confirmed prebiotic compounds, such as inulin (a natural soluble fiber) have an effect on microbiota composition as demonstrated in many studies including a study with 12 healthy volunteers investigating the effects of inulin consumption for 21 days on the microbiota where increases in the Bifidobacteria population were observed (58).

There were no changes in fasting insulin levels following the FWC supplementation, but inter-individual differences in response profiles existed. Insulin sensitivity could be more accurately studied with participants at risk of developing diabetes to study any long-term effects on glycaemic control. Previous data indicate that this benefit might be more pronounced in persons with a higher BMI. This was shown in a double-blind, randomized, placebo-controlled crossover study, where a daily dose of 20 g inulin-propionate ester was able to alter insulin sensitivity significantly (59).

There were several limitations associated with our study. There is increasing evidence that gut microbiota richness is linked to stool consistency and water content, which were not assessed in this study (60). This pilot trial was exploratory and thus did not use a control group or a cross over design with placebo treatment. As the study was conducted in a non-blinded manner, we cannot exclude treatment effect at least partially explaining the observed changes in the subjective GI well-being questionnaire. It is important to also bear in mind that the participants were free-living and were able to consume food *ad-libitum*, and diet is one of the key factors affecting microbiota diversity. Although in this study we collect 3-days food diaries to monitor changes in energy intake, it was not possible to assess changes in food intake patterns and food types consumed but macronutrient intake was fairly consistent at both the timepoints when diaries were completed. Weighed food diaries, as a dietary assessment method, have their limitations, and in our study also do not directly correspond with the timing of stool sample collection which is a further source of uncertainty (57).

We observed increased fecal propionate and butyrate concentrations following FWC consumption. The health effects of fecal butyrate can be attributed to the fact that colonocytes utilize bacterially-produced butyrate as their primary energy source, whereas most other cell types utilize glucose (61). Changes in gut permeability may improve gut health and confer long-term benefit to the host which we were unable to investigate in our short-term study. Changes in the microbiota can be attributed to a huge variety of factors including diet, immune challenges, exercise, variations in GI transit time, intestinal pH, host secretions, regulation of gene expression of the host and/or the microbiota and other environmental factors. Therefore, it is incredibly difficult to definitively attribute any changes in metabolic activity of the microbiota to the FWC supplementation. As highlighted by Stiemsma et al., compositional changes to the gut microbiota in addition to changes to immune or metabolic factors remain the most studied links between fermented food consumption and health outcomes (31). An increased focus on functional analyses, such as SCFA production, and the balance of SCFA production and absorption in humans as a result of fermented food consumption might shed light on their health benefits.

Probiotics and prebiotics are established dietary interventions seeking to correct underlying microbiota dysbiosis and leading ultimately to improved host health. However, a potentially new subtype of gut-directed supplements aiming to correct underlying microbiota dysbiosis in order to influence host health are post-biotics. The term post-biotic refers to “soluble factors (products or metabolic byproducts) secreted by live bacteria or released after bacterial lysis” (62). The key difference is that while probiotics, by definition, need to contain viable bacterial cells, post-biotics do not (28). Post-biotics must originate from bacteria, differentiating them from prebiotics, which tend to be fermentable carbohydrates. At present the few studies which have investigated the effectiveness of post-biotics indicate positive effects on host immune response both in animal and humans (62, 63).

This project was undertaken to evaluate the effect of bacterially derived FWC, with a high L-(+)-Lactic acid content, on microbiota composition and activity and determine potential effects on host health. Based on our data it is likely that the effects of FWC might be linked to the high lactic acid content specifically stimulating species of the resident microbiota. Consequently, we could consider FWC as a putative prebiotic based on the changes in microbial activity, or as a putative post-biotic since the lactic acid component is a product of the Lactobacillus fermentation of the whey, and is safe for human consumption at these concentrations. Confirmation of the FWC in either category would require establishment of a definitive health benefit. Future controlled human studies are required to establish our exploratory data in a larger participant group, with additional biological measures to establish the biodistribution of L-(+)-Lactic acid post-ingestion, and establish a health benefit. Taken together, this will help us understand if microbiota driven effects, such as the observed changes in SCFA profile, are directly beneficial to the host.

## Data Availability Statement

The datasets generated for this study can be found in online repositories. The names of the repository/repositories and accession number(s) can be found below: http://www.ebi.ac.uk/ena/data/view/PRJEB30495.

## Ethics Statement

The studies involving human participants were reviewed and approved by Research ethics committee of the Rowett Institute, University of Aberdeen (Reference Nr. 799). The patients/participants provided their written informed consent to participate in this study.

## Author Contributions

AJ, KS, and AS: conceptualization and funding acquisition. NM and JM: methodology. GH and SS: formal analysis. NS, NM, JM, and CF: investigation and resources by A.Vogel Bioforce AG. GH, SS, and NS: data curation. NS: writing-original draft preparation. AJ, KS, CF, and JM: writing-review and editing. NS and SS: visualization. AJ and KS: supervision. All authors contributed to the article and approved the submitted version.

## Conflict of Interest

AS was employed by the company Bioforce AG. A.Vogel Bioforce AG, AJ, and KS have conducted other contract research. The remaining authors declare that the research was conducted in the absence of any commercial or financial relationships that could be construed as a potential conflict of interest.

## References

[1] Valdes AM Walter J Segal E Spector TD. Role of the gut microbiota in nutrition and health. BMJ. (2018) 361:k2179. 10.1136/bmj.k2179 29899036 PMC6000740

[2] Flint HJ Duncan SH Scott KP Louis P. Links between diet, gut microbiota composition and gut metabolism. Proc Nutr Soc. (2015) 74:13–22. 10.1017/S0029665114001463 25268552

[3] Frieden TR. Evidence for health decision making—beyond randomized, controlled trials. N Engl J Med. (2017) 377:465–75. 10.1056/NEJMra1614394 28767357

[4] Martin CR Osadchiy V Kalani A Mayer EA. The brain-gut-microbiome axis. Cell Mol Gastroenterol Hepatol. (2018) 6:133–48. 10.1016/j.jcmgh.2018.04.003 30023410 PMC6047317

[5] de Vos WM de Vos EA. Role of the intestinal microbiome in health and disease: from correlation to causation. Nutr Rev. (2012) 70:S45–56. 10.1111/j.1753-4887.2012.00505.x 22861807

[6] Sanz Y Santacruz A Gauffin P. Gut microbiota in obesity and metabolic disorders. Proc Nutr Soc. (2010) 69:434–41. 10.1017/S0029665110001813 20540826

[7] Clemente JC Ursell LK Parfrey LW Knight R. The impact of the gut microbiota on human health: an integrative view. Cell. (2012) 148:1258–70. 10.1016/j.cell.2012.01.035 22424233 PMC5050011

[8] Dang D Zhou W Lun ZJ Mu X Wang DX Wu H Meta-analysis of probiotics and/or prebiotics for the prevention of eczema. J Int Med Res. (2013) 41:1426–36. 10.1177/0300060513493692 23908398

[9] Yoo JY Kim SS. Probiotics and prebiotics: present status and future perspectives on metabolic disorders. Nutrients. (2016) 8:173. 10.3390/nu8030173 26999199 PMC4808900

[10] Boyle RJ Tang ML-K Chiang WC Chua MC Ismail I Nauta A. Prebiotic-supplemented partially hydrolysed cow's milk formula for the prevention of eczema in high-risk infants: a randomized controlled trial. Allergy. (2016) 71:701–10. 10.1111/all.12848 27111273 PMC4996326

[11] Luoto R Ruuskanen O Waris M Kalliomäki M Salminen S Isolauri E. Prebiotic and probiotic supplementation prevents rhinovirus infections in preterm infants: a randomized, placebo-controlled trial. J Allergy Clin Immunol. (2014) 133:405–13. 10.1016/j.jaci.2013.08.020 24131826 PMC7112326

[12] Vulevic J Juric A Tzortzis G Gibson GR. A mixture of trans-galactooligosaccharides reduces markers of metabolic syndrome and modulates the fecal microbiota and immune function of overweight adults. J Nutr. (2013) 143:324–31. 10.3945/jn.112.166132 23303873

[13] Okubo H Nakatsu Y Kushiyama A Yamamotoya T Matsunaga Y Inoue M. Gut microbiota as a therapeutic target for metabolic disorders. Curr Med Chem. (2018) 25:984–1001. 10.2174/0929867324666171009121702 28990516

[14] Harris LA Baffy N. Modulation of the gut microbiota: a focus on treatments for irritable bowel syndrome. Postgrad Med. (2017) 129:872–88. 10.1080/00325481.2017.1383819 28936910

[15] Cani PD Neyrinck AM Fava F Knauf C Burcelin RG Tuohy KM. Selective increases of bifidobacteria in gut microflora improve high-fat-diet-induced diabetes in mice through a mechanism associated with endotoxaemia. Diabetologia. (2007) 50:2374–83. 10.1007/s00125-007-0791-0 17823788

[16] Tariq R Pardi DS Bartlett MG Khanna S. Low cure rates in controlled trials of fecal microbiota transplantation for recurrent *Clostridium difficile* infection: a systematic review and meta-analysis. Clin Infect Dis. (2019) 68:1351–8. 10.1093/cid/ciy721 30957161

[17] Chassard C Dapoigny M Scott KP Crouzet L Del'homme C Marquet P. Functional dysbiosis within the gut microbiota of patients with constipated-irritable bowel syndrome. Aliment Pharmacol Ther. (2012) 35:828–38. 10.1111/j.1365-2036.2012.05007.x 22315951

[18] Soret R Chevalier J De Coppet P Poupeau G Derkinderen P Segain JP. Short-chain fatty acids regulate the enteric neurons and control gastrointestinal motility in rats. Gastroenterology. (2010) 138:1772–82.e4. 10.1053/j.gastro.2010.01.053 20152836

[19] Louis P Flint HJ Michel C. How to manipulate the microbiota:prebiotics. Adv Exp Med Biol. (2016) 902:119–42. 10.1007/978-3-319-31248-4_9 27161355

[20] Scott KP Grimaldi R Cunningham M Sarbini SR Wijeyesekera A Tang ML. Developments in understanding and applying prebiotics in research and practice–an ISAPP conference paper. J Appl Microbiol. (2020) 128:934–49. 10.1111/jam.14424 31446668

[21] Macfarlane GT Steed H Macfarlane S. Bacterial metabolism and health-related effects of galacto-oligosaccharides and other prebiotics. J Appl Microbiol. (2008) 104:305–44. 10.1111/j.1365-2672.2007.03520.x 18215222

[22] Whelan K. Mechanisms and effectiveness of prebiotics in modifying the gastrointestinal microbiota for the management of digestive disorders. Proc Nutr Soc. (2013) 72:288–98. 10.1017/S0029665113001262 23680358

[23] Tzounis X Rodriguez-Mateos A Vulevic J Gibson GR Kwik-Uribe C Spencer JP. Prebiotic evaluation of cocoa-derived flavanols in healthy humans by using a randomized, controlled, double-blind, crossover intervention study. Am J Clin Nutr. (2011) 93:62–72. 10.3945/ajcn.110.000075 21068351

[24] Veiga P Pons N Agrawal A Oozeer R Guyonnet D Brazeilles R. Changes of the human gut microbiome induced by a fermented milk product. Sci Rep. (2014) 4:6328. 10.1038/srep06328 25209713 PMC4160712

[25] Fukumoto S Tatewaki M Yamada T Fujimiya M Mantyh C Voss M. Short-chain fatty acids stimulate colonic transit via intraluminal 5-HT release in rats. Am J Physiol Integr Comp Physiol. (2003) 284:R1269–76. 10.1152/ajpregu.00442.2002 12676748

[26] Vanhoutvin SALW Troostf FJ Kilkenst TOC Lindsey PJ Hamer HM Jonkers DMAE. The effects of butyrate enemas on visceral perception in healthy volunteers. Neurogastroenterol Motil. (2009) 21:952-e76. 10.1111/j.1365-2982.2009.01324.x 19460106

[27] Dimidi E Cox SR Rossi M Whelan K. Fermented foods:definitions and characteristics, impact on the gut microbiota and effects on gastrointestinal health and disease. Nutrients. (2019) 11:1806. 10.3390/nu11081806 31387262 PMC6723656

[28] Hill C Guarner F Reid G Gibson GR Merenstein DJ Pot B. The international scientific association for probiotics and prebiotics consensus statement on the scope and appropriate use of the term probiotic. Nat Rev Gastroenterol Hepatol. (2014) 11:506–14. 10.1038/nrgastro.2014.66 24912386

[29] Zhang C Derrien M Levenez F Brazeilles R Ballal SA Kim J. Ecological robustness of the gut microbiota in response to ingestion of transient food-borne microbes. ISME J. (2016) 10:2235–45. 10.1038/ismej.2016.13 26953599 PMC4989305

[30] Mättö J Fondén R Tolvanen T von Wright A Vilpponen-Salmela T Satokari R Intestinal survival and persistence of probiotic lactobacillus and bifidobacterium strains administered in triple-strain yoghurt. Int Dairy J. (2006) 16:1174–80. 10.1016/j.idairyj.2005.10.007

[31] Stiemsma LT Nakamura RE Nguyen JG Michels KB. Does consumption of fermented foods modify the human gut microbiota? J Nutr. (2020) 150:1680–92. 10.1093/jn/nxaa077 32232406 PMC7330458

[32] Eldridge SM Chan CL Campbell MJ Bond CM Hopewell S Thabane L. CONSORT 2010 statement: extension to randomised pilot and feasibility trials. BMJ. (2016) 355:i5239. 10.1136/bmj.i5239 27777223 PMC5076380

[33] Molkosan for Digestion|A Prebiotic Product for Healthy Digestion. Available online at: https://www.avogel.co.uk/food/products/molkosan-digestion/ (accessed March 26, 2018).

[34] Richardson AJ Calder AG Stewart CS Smith A Simultaneous determination of volatile and non-volatile acidic fermentation products of anaerobes by capillary gas chromatography. Lett Appl Microbiol. (1989) 9:5–8. 10.1111/j.1472-765X.1989.tb00278.x

[35] Andrews S Fast QC: A Quality Control Tool for High Throughput Sequence Data. (2015). Available online at: https://www.bioinformatics.babraham.ac.uk/projects/fastqc/ (accessed June 27, 2018).

[36] Callahan BJ Mc Murdie PJ Rosen MJ Han AW Johnson AJA Holmes SP. DADA2: high-resolution sample inference from Illumina amplicon data. Nat Methods. (2016) 13:581–3. 10.1038/nmeth.3869) 27214047 PMC4927377

[37] McDonald D Price MN Goodrich J Nawrocki EP De Santis TZ Probst A. An improved greengenes taxonomy with explicit ranks for ecological and evolutionary analyses of bacteria and archaea. ISME J. (2012) 6:610–8. 10.1038/ismej.2011.139 22134646 PMC3280142

[38] Mc Donald D Clemente JC Kuczynski J Rideout JR Stombaugh J Wendel D. The Biological Observation Matrix (BIOM) format or: how I learned to stop worrying and love the ome-ome. Gigascience. (2012) 1:7. 10.1186/2047-217X-1-7 23587224 PMC3626512

[39] Caporaso JG Kuczynski J Stombaugh J Bittinger K Bushman FD Costello EK. QIIME allows analysis of high-throughput community sequencing data. Nat Methods. (2010) 7:335–6. 10.1038/nmeth.f.303 20383131 PMC3156573

[40] Chao A Nonparametric estimation of the number of classes in a population. Scand J Stat. (1984) 11:265–70.

[41] Shannon CE A mathematical theory of communication. (2018) 27:379–423. 10.1002/j.1538-7305.1948.tb01338.x 9230594

[42] Simpson EH Measurement of diversity. Nature. (1949) 163:688 10.1038/163688a0

[43] Jaccard P The distribution of the flora in the alpine zone. New Phytol. (1912) 11:37–50. 10.1111/j.1469-8137.1912.tb05611.x

[44] Vázquez-Baeza Y Pirrung M Gonzalez A Knight R. EMPeror: a tool for visualizing high-throughput microbial community data. Gigascience. (2013) 2:16. 10.1186/2047-217X-2-16 24280061 PMC4076506

[45] Anderson MJ A new method for non-parametric multivariate analysis of variance. Austral Ecol. (2001) 26:32–46. 10.1046/j.1442-9993.2001.01070.x

[46] Martin BD Witten D Willis AD Modeling microbial abundances and dysbiosis with beta-binomial regression. arXiv. (2019) 1902.02776. 10.1214/19-aoas1283 PMC7514055 32983313

[47] Louis P Young P Holtrop G Flint HJ. Diversity of human colonic butyrate-producing bacteria revealed by analysis of the butyryl-Co A: acetate Co A-transferase gene. Environ Microbiol. (2010) 12:304–14. 10.1111/j.1462-2920.2009.02066.x 19807780

[48] Duncan SH Louis P Flint HJ. Lactate-utilizing bacteria, isolated from human feces, that produce butyrate as a major fermentation product. Appl Environ Microbiol. (2004) 70:5810–7. 10.1128/AEM.70.10.5810-5817.2004 15466518 PMC522113

[49] Bourriaud C Robins RJ Martin L Kozlowski F Tenailleau E Cherbut C. Lactate is mainly fermented to butyrate by human intestinal microfloras but inter-individual variation is evident. J Appl Microbiol. (2005) 99:201–12. 10.1111/j.1365-2672.2005.02605.x 15960680

[50] Stumpff F. A look at the smelly side of physiology: transport of short chain fatty acids. Pflügers Arch Eur J Physiol. (2018) 470:571–98. 10.1007/s00424-017-2105-9 29305650

[51] Cecilia Riegel B The rate of disappearance of sodium lactate injected intravenously and its effect upon sugar and inorganic phosphate of the blood. J Biol Chem. (1927) 74:135–48.

[52] Philp A Macdonald AL Watt PW Weibel ER Taylor CR. Lactate–a signal coordinating cell and systemic function. J Exp Biol. (2005) 208:4561–75. 10.1242/jeb.01961 16326938

[53] Morotomi M Sakai K Yazawa K Suegara N Kawai Y Mutai M. Effect and fate of orally administered lactic acid in rats. J Nutr Sci Vitaminol. (1981) 27:117–28. 10.3177/jnsv.27.117 7310549

[54] Duncan SH Belenguer A Holtrop G Johnstone AM Flint HJ Lobley GE. Reduced dietary intake of carbohydrates by obese subjects results in decreased concentrations of butyrate and butyrate-producing bacteria in feces. Appl Environ Microbiol. (2007) 73:1073–8. 10.1128/AEM.02340-06 17189447 PMC1828662

[55] Phypers B Pierce JT Lactate physiology in health and disease. Contin Educ Anaesth Crit Care Pain. (2006) 6:128–32. 10.1093/bjaceaccp/mkl018

[56] Scheiman J Luber JM Chavkin TA Mac Donald T Tung A Pham L-D. Meta-omics analysis of elite athletes identifies a performance-enhancing microbe that functions via lactate metabolism. Nat Med. (2019) 25:1104–9. 10.1038/s41591-019-0485-4 31235964 PMC7368972

[57] Bingham SA Gill C Welch A Day K Cassidy A Khaw KT. Comparison of dietary assessment methods in nutritional epidemiology: weighed records v. 24 h recalls, food-frequency questionnaires and estimated-diet records. Br J Nutr. (1994) 72:619–43. 10.1079/BJN19940064 7986792

[58] Fuller Z Louis P Mihajlovski A Rungapamestry V Ratcliffe B Duncan AJ. Influence of cabbage processing methods and prebiotic manipulationof colonic microflora on glucosinolate breakdown in man. Br J Nutr. (2007) 98:364. 10.1017/S0007114507709091 17403273

[59] Chambers ES Byrne CS Morrison DJ Murphy KG Preston T Tedford C. Dietary supplementation with inulin-propionate ester or inulin improves insulin sensitivity in adults with overweight and obesity with distinct effects on the gut microbiota, plasma metabolome and systemic inflammatory responses: a randomised cross-over trial. Gut. (2019) 68:1430–8. 10.1136/gutjnl-2019-318424 30971437 PMC6691855

[60] Falony G Vieira-Silva S Raes J. Richness and ecosystem development across faecal snapshots of the gut microbiota. Nat Microbiol. (2018)3:526–8. 10.1038/s41564-018-0143-5 29693658

[61] Donohoe DR Garge N Zhang X Sun W O'Connell TM Bunger MK. The microbiome and butyrate regulate energy metabolism and autophagy in the mammalian colon. Cell Metab. (2011) 13:517–26. 10.1016/j.cmet.2011.02.018 21531334 PMC3099420

[62] Aguilar-Toalá JE Garcia-Varela R Garcia HS Mata-Haro V González-Córdova AF Vallejo-Cordoba B Postbiotics: an evolving term within the functional foods field. Trends Food Sci Technol. (2018) 75:105–14. 10.1016/j.tifs.2018.03.009

[63] Tsilingiri K Rescigno M. Postbiotics: what else? Benef Microbes. (2013) 4:101–7. 10.3920/BM2012.0046 23271068

