# Supplementary material for: Daily Fermented Whey Consumption Alters the Fecal Short-Chain Fatty Acid Profile in Healthy Adults
Source: Front Nutr. 2020 Sep 30;7:165. doi: 10.3389/fnut.2020.00165 (PMC7556162; doi:10.3389/fnut.2020.00165)
Supplement: Supplementary file 1 [file Data_Sheet_1.docx]

**Supplementary material:**

**Table S1.** Differences in the bacterial composition of individuals with the smallest and largest relative changes in propionate and butyrate following FWC consumption, as determined by analysis with Corncob. FDR: false discovery rate correction for multiple testing.

1. **Propionate**

Non responders ID: 21, 1, 13, 19, 22

Responder ID: 14, 4, 16, 17, 12

All time points grouped together:

| **Taxonomic level** |  | **Higher in** | **FDR** |
| --- | --- | --- | --- |
| Family | *Bacteroidaceae* | Non-responder | 0.024 |
| Genus | *Bacteroides* | Non-responder | 0.0095 |
|  | *Lachnospiraceae; NA.* | Responder | 0.026 |
|  | *Coprococcus* | Non-responder | 0.0019 |
|  | *Alistipes* | Responder | 0.043 |
|  | *Collinsella* | Responder | 0.00053 |
|  | *Oscillospira* | Non-responder | 0.043 |
|  | *Phascolarctobacterium* | Non-responder | 0.00000113 |
|  | *Butyricicoccus* | Non-responder | 0.000060 |
| Species | *Bacteroides uniformis* | Non-responder | 0.0026 |
|  | *Lachnospiraceae_NA_NA.* | Responder | 0.044 |
|  | *Coprococcus; s__.* | Non-responder | 0.0021 |
|  | *Phascolarctobacterium; s__.* | Non-responder | 0.00000158 |
|  | *Butyricicoccus pullicaecorum* | Non-responder | 0.000084 |

T1 Responder vs. Non-responder:

| **Taxonomic level** |  | **Higher in** | **FDR** |
| --- | --- | --- | --- |
| Genus | *Phascolarctobacterium* | Non-responder | 0.029 |
|  | *Butyricicoccus* | Responder | 0.017 |
| Species | *Phascolarctobacterium sp.* | Non-responder | 0.040 |
|  | *Butyricicoccus pullicaecorum* | Responder | 0.024 |

T3 Responder vs. Non-responder:

| **Taxonomic level** |  | **Higher in** | **FDR** |
| --- | --- | --- | --- |
| Genus | *Phascolarctobacterium* | Non-responder | 0.011 |
| Species | *Phascolarctobacterium sp.* | Non-responder | 0.016 |

T6 Responder vs Non-responder – no differences

1. **Butyrate**

Non-responders ID: 7, 15, 11, 21, 6

Responders ID: 18, 4, 14, 20, 12

| **Taxonomic level** |  | **Higher in** | **FDR** |
| --- | --- | --- | --- |
| Phylum | *Verrucomicrobia* | Non-responder | 0.029 |
| Family | *Veillonellaceae* | Responder | 0.0079 |
| Genus | *Dialister* | Responder | 0.0071 |
|  | *[Eubacterium]* | Responder | 0.010 |
| Species | *Bacteroides uniformis* | Non-Responder | 0.0071 |
|  | *Ruminococcus bromii* | Non-Responder | 0.010 |
|  | *Dialister sp.* | Responder | 0.0047 |
|  | *Bacteroides eggerthii* | Responder | 0.038 |

All time points grouped together:

T6 Responder vs. Non-responder:

| **Taxonomic level** |  | **Higher in** | **FDR** |
| --- | --- | --- | --- |
| Phylum | *Verrucomicrobia* | Non-responder | 0.0067 |
| Family | *Verrucomicrobiaceae* | Non-Responder | 0.033 |

All other time point comparisons had non-significant findings

**Table S2.** SCFA composition of fermented whey concentrate in absolute and relative terms.

Two separate samples, from same batch of FWC, were analysed in duplicate as described in section 2.8. The SCFA profile is dominated by the high lactic acid concentration (93.39%). No valerate, iso-valerate, iso-butyrate or fumarate were detected.

|  | **Formate** | **Acetate** | **Propionate** | **Butyrate** | **Lactate** | **Succinate** | **Total SCFA** |
| --- | --- | --- | --- | --- | --- | --- | --- |
| **Average (mM)** | 6.46 | 40.06 | 0.46 | 0.04 | 673.74 | 0.68 | 721.44 |
| **SD** | 0.23 | 1.18 | 0.08 | 0.04 | 17.14 | 0.68 |  |
| **% total** | 0.90 | 5.55 | 0.06 | 0.01 | 93.39 | 0.09 |  |
